# Artificial intelligence in the pre-analytical phase: State-of-the art and future perspectives

**DOI:** 10.5937/jomb0-45936

**Published:** 2024-01-25

**Authors:** Giuseppe Lippi, Camilla Mattiuzzi, Emmanuel J. Favaloro

**Affiliations:** 1 University of Verona, Section of Clinical Biochemistry and School of Medicine, Verona, Italy; 2 Hospital of Rovereto, Provincial Agency for Social and Sanitary Services (APSS), Medical Direction, Trento, Italy; 3 Institute of Clinical Pathology and Medical Research (ICPMR), Sydney Centres for Thrombosis and Haemostasis, Department of Haematology, NSW Health Pathology, Westmead Hospital, Westmead, NSW Australia; 4 University of Sydney, Faculty of Medicine and Health, School of Medical Sciences, Westmead Hospital, Westmead, New South Wales, Australia + Charles Sturt University, Faculty of Science and Health, Wagga Wagga, NSW, Australia

**Keywords:** artificial intelligence, robotics, preanalytical phase, preanalytical variability, errors, veštačka inteligencija, robotika, preanalitička faza, preanalitička varijabilnost, greške

## Abstract

The use of artificial intelligence (AI) has become widespread in many areas of science and medicine, including laboratory medicine. Although it seems obvious that the analytical and post-analytical phases could be the most important fields of application in laboratory medicine, a kaleidoscope of new opportunities has emerged to extend the benefits of AI to many manual labor-intensive activities belonging to the pre-analytical phase, which are inherently characterized by enhanced vulnerability and higher risk of errors. These potential applications involve increasing the appropriateness of test prescription (with computerized physician order entry or demand management tools), improved specimen collection (using active patient recognition, automated specimen labeling, vein recognition and blood collection assistance, along with automated blood drawing), more efficient sample transportation (facilitated by the use of pneumatic transport systems or drones, and monitored with smart blood tubes or data loggers), systematic evaluation of sample quality (by measuring serum indices, fill volume or for detecting sample clotting), as well as error detection and analysis. Therefore, this opinion paper aims to discuss the state-of-the-art and some future possibilities of AI in the preanalytical phase.

## Abstract (sr)

Upotreba veštačke inteligencije (AI) je postala široko rasprostranjena u mnogim oblastima nauke i medicine, uključujući laboratorijsku medicinu. Iako se čini očiglednim da bi analitička i postanalitička faza mogle da budu najvažnija polja primene u laboratorijskoj medicini, pojavio se kaleidoskop novih mogućnosti za proširenje prednosti veštačke inteligencije na mnoge aktivnosti ručnog rada koje pripadaju preanalitičkoj fazi, koje karakteriše povećana ranjivost i veći rizik od grešaka. Ove potencijalne aplikacije uključuju povećanje prikladnosti propisivanja testova (sa kompjuterizovanim unosom naloga lekara ili alatima za upravljanje potražnjom), poboljšano prikupljanje uzoraka (koristeći aktivno prepoznavanje pacijenata, automatsko obeležavanje uzoraka, prepoznavanje vena i pomoć pri prikupljanju krvi, zajedno sa automatskim vađenjem krvi), više efikasan transport uzoraka (omogućen upotrebom pneumatskih transportnih sistema ili dronova, i praćen pametnim epruvetama za krv ili snimačima podataka), sistematsko ocenjivanje kvaliteta uzorka (merenjem indeksa seruma, zapremine punjenja ili za otkrivanje zgrušavanja uzorka), kao i greške otkrivanje i analizu. Stoga, ovaj rad ima za cilj da diskutuje o stanju tehnike i nekim budućim mogućnostima AI u preanalitičkoj fazi.

## Introduction

The term artificial intelligence (AI) is commonly used, according to the Oxford Dictionary, as the study and development of computer systems that can mimic intelligent human behavior [1]. AI, then, is not a new topic, as the term was first introduced in 1956 and encompasses the development of computer systems capable of efficiently performing tasks that normally require human intelligence [2].

The use of AI has now become commonplace in most areas of science and medicine, including laboratory medicine [3] [4]. Although it may seem obvious that the most important application areas of laboratory medicine would be the analytical phase (e.g., calculation of ratios or fractions between different test results, reflex testing, diagnostic algorithms, etc.) or the post-analytical phase (e.g., automated generation of interpretative comments, laboratory data integration, auto-validation and so forth), a kaleidoscope of new opportunities has emerged to extend the benefits of AI to many manually-intensive activities of the pre-analytical phase, which are highly vulnerable and thus characterized by higher risk of errors [5]. More specifically, intelligent systems together with robotics offer the opportunity to automate several laboratory activities, from test ordering to assessment of sample quality, thus optimizing the workflow, minimizing the risk of errors, and increasing test throughput, ultimately producing a high degree of laboratory efficiency and a faster turnaround time. These potential solutions may be stand-alone solutions to overcome specific problems, or may be incorporated into laboratory or hospital information systems as ’expert rules’ [6]. This opinion paper is hence devoted to discussing the state-of-the-art and some future opportunities of AI in the pre-analytical phase, as summarized in Table 1.

**Table 1:** Opportunities for artificial intelligence (AI) and robotics AI in the pre-analytical phase.

• Appropriateness of test prescription
o Computerized (Physician) Order entry
o Demand Management Tools
• Sample collection
o Positive patient recognition
o Automated sample labeling
o Vein detection and blood draw assistance
o Automated sample collection
• Sample transportation
o Pneumatic transport systems
o Drones
o Smart blood tubes
o Data loggers
• Automated assessment of sample quality
o Hemolysis indices
o Clot detection
o Fill volume
• Error recording and analysis

### Appropriateness of test prescription

Test ordering can reasonably be considered one of the most critical components of the total testing process, since it basically relies on the so-called »R paradigm« (i.e., performing the Right test, with the Right method, at the Right time, to the Right patient, at the Right cost, for the Right outcome) [7]. Nonetheless, several lines of evidence garnered over the past decades suggest that inappropriateness of laboratory test ordering is still the big elephant in the room, with up to 70% of total tests potentially inappropriately ordered for a variety of different reasons [8]. Therefore, whatever types of interventions that can be tailored to enhance appropriateness of requesting tests in the local reality has a high likelihood of reducing costs, providing more-patient-centric care and enhancing patient safety [9]. We review here some possible strategies based on intelligent systems that may be associated with enhanced appropriateness and/or patient safety.

### Computerized (physician) order entry

The Computerized (Physician) Order Entry (CPOE) encompass a number of computer-based software programs developed for automating the ordering process of medical interventions including drugs and/or diagnostic tests [10]. The advantages of using these computer-based systems (which can be adapted for use in personal computer, laptops, tablets and even mobile phones), include the generation of faster, unique (e.g., preventing requesting the same test twice), more standardized, intelligible and permanent orders, since these could be perpetually storedwithin the laboratory information system (LIS). Moreover, the automatic bi-directional connection between the LIS and the software of the laboratory analyzer (with or without an interposed middleware) would then enable the direct transfer of the test order to the analyzer and the performance of the requested test(s) once the barcoded sample is loaded. In a forthcoming virtual reality, the test order by the requesting physician from his/her personal device would allow automatic printing of the tube label, and automatic blood collection, which is then directly delivered to the analyzer without any intermediate intelligent human activity (which may only come afterwards, e.g., for test validation). Direct physician ordering also permits a means to input any relevant clinical information, as well as medications that may affect laboratory tests. For example, patients may be on a variety of anticoagulant medications to manage a recent thrombosis, and these may affect coagulation tests, or indeed may point to the reason for the order (i.e., anticoagulant monitoring) [11].

### Demand management tools

The so-called Demand Management Tools (DMTs) are information technology-based software programs that are typically included in, or connected with the CPOE. They frequently use patient-specific features, matching them with knowledge base by means of rule-based algorithms. In practice, there are many types of such tools, with different functions. Thus, they can produce specific reminders or recommendations with the purpose of influencing the prescription habit, they may limit the number and type of tests offered by using ordering algorithms or expert systems that assist the physician in selecting the more appropriate tests for a given clinical conditions, or can also be based on predefined alerts constructing according to test-specific (re-)testing intervals or using various gate-keeping strategies that do not allow the free request of one or more tests (the description of these digital instruments is summarized in Table 2). The rules used in both strategies can be locally customized to meet the specific organization of the laboratory and of the healthcare facilities served by the laboratory. Moreover, the inhibition of the test request can be irreversible (i.e., will not allow the ordering physician to move forward), or flexible, in that the alert could be overcome – for example – with verbal agreement with the laboratory or by entering a specific comment that explains why the rule could be violated.

**Table 2:** Some examples of Demand Management Tools (DMTs).

Tools	Description	Examples
Re-testing intervals	Impossibility to freely ordering laboratory tests<br>due to violation of a minimum time passed<br>between one request and another.	o Prescribing total cholesterol every day<br>o Assessing HbA1c every week
Gate-keeping strategy	Impossibility to freely ordering some laboratory<br>tests due to violation of a predefined set of prescription<br>rules.	o Prescribing prostate specific antigen (PSA) in<br>women<br>o Ordering free PSA when total PSA is normal<br>o Cancellation of direct bilirubin when total<br>bilirubin is normal

The use of these tools has recently been reviewed by Cadamuro [9] and Carobene [12], who concluded that DMTs may be a practical and cost-effective approach for better management of inappropriate usage of laboratory resources. Some specific data can also be provided in support of this statement. For example, in a seminal work published by one of us nearly 10 years ago, we showed that the adoption of a computerized alert system based on specific re-testing intervals and encompassing the appearance of flexible alerts (i.e., ignorable pop-ups) when a set of predefined appropriateness criteria were violated by the prescribing physicians, was effective to generate a nearly 80% reduction in the burden of theoretically inappropriate tests, accompanied by an approximately 13% decrease of total laboratory costs [13]. In another experience, Delvaux et al. [14]developed a clinical decision support systems (CDSS) encompassing a series of evidence-based order sets aimed at proposing the more appropriate lab tests according to the clinical indications provided by the prescribing physician. The authors showed that such system not only was effective to significantly enhance the number of appropriately requested tests by over 50%, but was also associated with significant decrease in potential diagnostic errors. Another interesting experience was published by Kumar et al. [15]. In brief, the authors developed a predefined set of rules in the order-entry that foresaw the automatic cancellation of some tests when other related exams were normal (i.e., cancellation of aspartate aminotransferase (AST) when alanine aminotransferase (ALT) was normal; cancellation of direct bilirubin when total bilirubin was normal; cancellation of free prostate specific antigen (PSA) when total PSA was normal), generating a saving of 78% tests requests for AST, 77% for direct bilirubin, and 72% for free PSA, estimating also a dramatic reduction of unnecessary medical treatments.

### Sample collection

Sample collection is, without doubts, the most critical part of the total testing process, i.e., the part of the so-called brain-to-brain loop that display the highest vulnerability to errors [16]. It is hence predictable that adoption of AI and/or robotics aids in this essential part of in vitro diagnostic testing could be associated with the best revenues in terms of efficiency and patient safety [17].

### Positive patient recognition

Patient (mis)identification has been for long a well-known source of diagnostic errors, since patient and/or sample misidentification can generate a vast array of dramatic health consequences [18]. Biometrics can be simply defined as recognition of humans based on individual-specific physical and behavioral characteristics. Although a variety of biometrical approaches can be used for human identification and/or authentication, the most commonly used involve fingerprint, palm (vein distribution), face, voice and eye (i.e., iris) recognition [19]. Irrespective of the strategy used for biometric recognition, the functionality is similar, involving a sequence of steps encompassing biometrical data acquisition, pre-processing, feature extraction, classification, evaluation and decision. Each of these strategies have their own advantages and limitations. Regarding performance, the currently available systems for palm vein (<0.1% false recognition rate; FRR), eye (0.2% FRR) and fingerprint (1.0% FRR) recognition enable the highest accuracy, followed by voice (3.0% FRR) and face (6.0% FRR) recognition [20]. On the other hand, the implementation, practical application and costs of such systems seems higher for face, palm and voice recognition, whilst fingerprint and eye recognition are usually more challenging to be adopted. As earlier mentioned,many aspects concur for selecting a particular approach within a healthcare setting, including the environment, local regulations and requirements, circumstances of application, acceptability and practicability. Overall, the application of biometrics in healthcare is currently limited to a very modest number of facilities, since more traditional strategies (especially identity cards for outpatients and barcoded wristband for inpatients) are still commonplace for recording patient identity. Nevertheless, the continuous technological evolution and the increasing emphasis given to privacy, data security and patient safety will certainly foster a predictable growth of these biometric applications for patient identification in healthcare.

### Automated sample labeling

All diagnostic specimens conveyed to diagnostic facilities must have a unique identifier, typically a barcoded label, which stores several information about patient identity and the types of tests to be performed. Although the question of whether specimens must be labeled before or after collection has not yet been resolved [21] [22], recent technological advances have made it possible to develop a wide range of devices for automatic and fully traceable labeling of containers used for collecting biological specimens [23]. These systems share a common mode of operation that includes a bilateral interface with the LIS to allow positive patient identification, order verification, and automatic tube selection and labelling, followed by automated check-out after sample collection has been completed. Several field reports have already been published on the many potential benefits that these devices can offer to tube labelling efficiency and safety. A comprehensive evaluation of two of such automatic sample labeling systems has been carried out by Piva et al. [24], using ProTube from Inpeco and ROBO from Becton Dickinson. The blood sampling procedure took around 3 min with ProTube and 5 min with ROBO, with a mean number of managed patients of 16 and 10 per hour, respectively. Importantly, no incorrectly or erroneously labeled tube could be identified throughout the evaluation period with either system. Lippi et al. [25] also conducted a retrospective investigation to test the performance of Inpeco ProTube at a large phlebotomy center. Compared to the pre-implementation period, the use of the automatic labeling device was associated with a remarkable reduction of wrong labeled tubes (-60%), samples lost (-52%) and underfilled tubes (-51%). No misidentification error occurred before and after implementation. Thus, these two studies would actually confirm that automatic sample labeling devices are fast, efficient, accurate and provide a potentially safer alternative to manual sample labelling.

### Vein detection and blood draw assistance

Locating a suitable vein to be punctured is a necessary prerequisite for blood collection. This procedure is not always easy, as the superficial vein tree may not be immediately visible (or may even be absent) and/or the operator may not have sufficient experience to accurately identify a puncturable vein [26]. Therefore, transilluminator devices have been specifically developed to facilitate this activity. They essentially consist of cold near-infrared light-emitting diodes (LEDs) whose light is absorbed by the intra-erythrocytic hemoglobin flowing in the veins, facilitating the identification of a suitable vessel to be punctured [27]. The effectiveness of these devices has been summarized in a meta-analysis of seven trials [28], which concluded that transilluminators were effective to improve by nearly 4-fold (odds ratio (OR), 3.96; 95%CI, 1.75–8.94) the venipuncture success rates compared to traditional drawing techniques. Importantly, the use of these devices did not prolong the time to cannulation, or increase the number of venipunctures needed for drawing blood. A second meta-analysis has also been published by Firooz et al. [29], which included four randomized control studies in children. Overall, the use of transilluminator devices increased by 34% (relative risk (RR), 1.34; 95%CI, 1.18–1.53) the success of peripheral venous catheter placement.

### Automated sample collection

Important advancements in robotics and medical technology are substantially reshaping the landscape of managed care, and one striking example is the development of automated sample collection devices. The use of robotics in healthcare is certainly not new, wherein their usage has become very common in surgery [30]. It is hence not surprising that many public and private companies have been actively involved in projecting specific robots for drawing blood. In practice,the research on prototypes of blood-drawing robots has commenced two decades ago [31], with development of devices accurately engineered for replicating the drawing technique of a skilled phlebotomist. These blood drawing robots are typically equipped with a combination of advanced sensors and imaging technology that allow to detect a suitable vein to be punctured, rapidly assessing its width and depth [32]. These characteristics may be theoretically apt to enhance the success rate of blood draws, limiting the risk of potential complications like pain, bruising or even vein injury. Predictably, the implementation of automated sample collection in healthcare may generate numerous benefits, such as streamlining and harmonizing blood collection and saving human resources that could be used for other healthcare activities. Nonetheless, all that glitters is not gold, at least at this point in time. Irrespective of the potential advantages and unlike surgical robots (which still basically need a human »pilot«), robots for drawing blood still need an extensive validation before they can be introduced into routine clinical practice. A number of studies have been published about their high efficiency in simulated venipuncture, though investigation in humans remains very limited to the best of our knowledge. To cite some recent examples, He et al. developed a six-degree-of-freedom venipuncture robot, used to collect blood from rabbit ear veins, and reported a success rate of approximately 90% [33]. In another study He et al. [34], refined their blood-drawing robot with decoupled position and attitude, consisting of near-infrared vision and laser sensors for garnering information on puncture site, 3-degree-of-freedom positioning manipulator for needle location and 3-degree-of-freedom end-effector for adjusting yaw and pitch angles of the needle. In 30 consecutive experiments, the venipuncture robot could always locate the exact puncture point in a phantom, and the overall time needed to conclude the venipuncture was around 20 seconds. Regarding studies involving human subjects, we could only identify one published by Leipheimer et al. [35]. The authors developed a robotic venipuncture device combining ultrasound imaging for identifying a suitable vein to be punctured coupled with miniaturized robotics equipped with a blood draw needle. A preliminary evaluation of this device in humans showed performance comparable to, or even better than that of human blood drawing. In particular, the success rate was 87%, increasing to 97% in patients with easier venous accesses, and with an average venipuncture time of around 90 seconds. Importantly, new devices are also emerging that combine image-guided venipuncture with discrete laboratory analyzers, which would hence enable near-patient automated blood drawing and point-of-care testing [36].

Besides robotics, semiautomated self-microsampling devices have also been recently commercialized. The construction and instruction for use of many of these devices are similar. Basically, the device can be attached to an upper arm through an adhesive and is then activated by (hand) pressure. The activation involves a relatively painless skin puncture, after which the vacuum applied by the device enables to automatically draw blood into an attached blood tube. The typical collection volume is between 0.1–0.5 mL and multiple (replaceable) types of blood tubes can be used. According to recent evaluations of some of these devices, patients indicate that blood drawing using these self-microsampling tools enabled more convenient blood drawings compared with finger-pricking and test results were comparable to those obtained with a standard blood collection technique [37] [38].

It is also worth noting that AI and robotics applications for specimen collection can go far beyond blood collection. Some prototype robots for throat or nasal swabs are already available [39] [40] [41] [42]. These automated swab machines are equipped with vision systems that can detect facial structure and identify the correct locations for swabbing the throat or nasopharynx. Basically, the arm of the robot collects the material by placing the swab securely in thenasopharynx or pharynx for an appropriate amount of time, then placing it in a jar and unscrewing the lid. In this way, the risk of cross-infection can be reduced and valuable time and resources saved, as the entire swabbing procedure can be completed in 5 minutes, compared to the 10–15 min that would be required for a traditional procedure performed by medical personnel. However, as with blood collection robots, the lack of clinical validation studies remains a major drawback.

### Sample transportation

Regardless of the location of the diagnostic facility where specimens must be delivered for testing, and with the exception of near-patient testing (i.e., point-of-care testing), specimen transport is always an important part of the overall testing process and is also an activity where some preanalytical errors can occur (e.g., specimens that are lost, damaged, or transported in inappropriate environmental conditions) [42]. The development and implementation of reliable means and validated strategies to protect specimens during transport must therefore be considered a high priority in laboratory medicine.

### Pneumatic transport systems

Pneumatic transport systems (PTSs) are indeed one of the most widespread tools for delivering samples and other materials (e.g., blood bags, documents) within healthcare facilities [43]. These systems encompass the generation of an air pressure for moving sealed containers (carriers) through a network of pneumatic tubes, thus enabling a rapid and highly efficient transfer of biospecimens. The obvious advantages of these systems include saving human resources for transportation, speed, efficiency, low risk of contamination and loss of samples, possible integration with the LIS, tracking and traceability.

Unlike earlier models, where acceleration, speed and deceleration were so critical that the risk ofdamaging blood was unacceptably high, the new generation of these devices has allowed safer transportation, as shown in the meta-analysis published by Nybo and co-authors [44] and some following studies [45]. Nevertheless, it is still advisable that – due to the high heterogeneity of the solutions available in the market – clinical laboratories will need to locally validate the correct functioning of their systems, for example by assaying acceleration forces and comparing the quality of test results in samples conveyed by PTS or manually transported.

### Drones

Drones have recently emerged as potential alternatives to PTS for short- or medium-distance shipping of biospecimens. Drones can be simply defined as »flying robots« or »unmanned air vehicles«, which could be remotely controlled or could fly autonomously using GPS (global positioning system) and software-controlled flight plans [46]. The many possible advantages of flying samples with drones include rapid transportation with active control from remote distance, the possibility to reach distant and/or isolated areas that could be difficult to access with traditional transportation, environmental benefits, lower transportation costs, real-time tracking and monitoring, along with scalability. On the other hand, there are also some key aspects that must be considered before deciding to implement drone transportation for delivering biospecimens, such as regulatory approval, safety considerations (e.g., crashes or risk of incidents with other flying vehicles), privacy concerns, robust infrastructure to support flying operations, limited transportable weight, dependence of flying on environmental conditions and need to adopt reliable systems for safeguarding sample quality [46]. Another crucial and virtually philosophical aspect is that we will need to enhance our real understanding on why, when, and how would be actually required to use drones or other aids for off-site transport for purposes of consolidating laboratory services [47].

### Smart blood tubes

The so-called »smart blood tubes«, also known as »chip-enabled tubes«, are blood tubes containing integrated microchips, thus representing another emerging technology in the enterprise of blood drawing and medical diagnostics [48]. Several examples of these new containers have been proposed, such as those presented by El Khamlichi et al. [49] or by Caredda et al. [50]. These devices have also many potential advantages, such as direct data integration with the LIS, and possibility to avoid the use of barcoded sample labels for storing demographic and testing information, better identification and tracking of samples, improved data accuracy, real-time continuous monitoring of sample location. Nonetheless, some important aspects would need to be analyzed before widespread usage of these devices, including manufacturing costs, data security, reliability of technology and regulatory approval.

At least theoretically, it is then possible to develop the so-called »Lab-in-a-tube« (i.e., integration of laboratory functions within a chip inserted into the tube) [51]. Although the current applications of these devices are still limited, the remarkable advancements in microfluidics, miniaturized analytics and lab-on-a-chip technology will predictably boost this part of the diagnostic industry in the foreseeable future [52], especially for rapid and decentralized detection of human pathogens (including SARS-CoV-2) [53].

### Data loggers

Data loggers, often used as mandatory components of biospecimen transportation, are devices that have been developed for monitoring and recording several environmental parameters and conditions throughout the process of sample delivery from the collection to the testing centers [54]. The data logger is activated after inserting the samples within the transport boxes and records a number of variables during transportation, typically including temperature, humidity, transportation time, sample location and, occasionally, shock, vibrations and light exposure. The use of data loggers during transportation of biospecimens would hence enable to garner a thoughtful picture of the environmental conditions to which samples have been exposed, with inherent advantages in terms of testing quality and patient safety.

### Automated assessment of sample quality

The systematic assessment of sample quality has now become a cornerstone of a total quality system in laboratory medicine, in that the presence of high levels of some specific interfering substances (namely cell-free hemoglobin, bilirubin, lipids) in serum or plasma may seriously jeopardize the reliability of some laboratory tests. The development and implementation of automated serum indices has virtually revolutionized the approach to assess sample quality, based for decades on the simple visual inspection [55]. Although a more comprehensive description of these quality tools has been provided in many prior articles [56] [57] [58], a brief narration may be worthwhile here as well. The so-called serum (or plasma) indices are fully-automated parameters used by many laboratory analyzers for assessing sample quality and, in particular, for identifying potentially interfering amounts of substances which that may ultimately impair the accuracy of test result. Thus, the serum indices typically comprise the hemolysis index (HI), the icteric index (II) and the lipemic index (LI). The estimation of these indices is based on measuring the concentration of the respective interfering substances (e.g., cell-free hemoglobin for HI, bilirubin for II, and lipids for LI, respectively) in the test sample with proprietary spectrophotometric tests. Basically, the analyzers measure and integrate light absorbance at instrument-specific wavelengths to quantify the degree of interference, providing then a semi-quantitative (i.e., in arbitrary units) or quantitative (i.e., as analyte concentration) measure of the interfering substance, which can be compared to predefined acceptability criteria that have been set for different interfe rencevulnerable tests (a typical example is a high HI that may trigger the suppression of potassium). Importantly, different analyzers use different wave-length, algorithms and cutoff values for estimating the interference risk, so that they are not straightforwardly comparable [59].

### Sample filling

An additional evaluation of sample volume has now also been integrated into some instruments. Insufficient filling of primary collection tubes is a long-known issue for coagulation tests [60], and has been more recently highlighted for blood counting [61].

### Sample clotting detection

Undue clotting, i.e., the formation of blood clots and/or platelet aggregates when this is not required or advisable (e.g., for blood cell counting, or for coagulation tests) is a relatively rare (i.e., around 5% of all rejected samples) but analytically and clinically important circumstance [5]. The presence of clots within the specimen impairs its cellular and biochemical composition, consequently biasing the values of several analytes, decreasing the analytical quality and jeopardizing patient’s health [62]. Failure patterns sensors have been specifically developed within the modern laboratory analyzers for reliably identifying the presence of clots in the diagnostic sample, the technical principles of which have been comprehensively reviewed elsewhere [63]. Nonetheless, additional approaches, based on intelligent software programs, have been suggested. For example, Fang et al. used a machine learning approach for identifying the potential presence of clots in plasma specimens [64]. In their hands, the integration of the test results of six different hemostasis tests enabled an accurate prediction (with 97% accuracy, 0.94 sensitivity and 0.97 specificity) of diagnostic samples containing blood clots.

### Error recording and analysis

Identification, recording and analysis of diagnostic errors are essential parts of a total quality management system [65]. Unfortunately, this practice is often considered time-consuming and even potentially useless by many laboratory professionals, thus hampering its widespread diffusion. As recently reported by the Working Group for the Preanalytical Phase (WG-PRE) of the European Federation of Clinical Chemistry and Laboratory Medicine (EFLM) [66], more than half of the clinical laboratories surveyed were not active in systematically analyzing laboratory errors, while nearly one-third had not set a process for follow-up and acting when preanalytical indicators exceeded their specific thresholds. Thus, designing informatic platforms that may facilitate and accelerate this procedure are an attractive and important solution. Specifically for this purpose, the EFLM WG-PRE and the Working Group on »Laboratory Errors and Patient Safety« (WG-LEPS) of the International Federation of Clinical Chemistry and Laboratory Medicine (IFCC) have jointly developed a software for recording preanalytical errors according to standardized quality indicators (QIs) endorsed by the project of QIs in Laboratory Medicine [67]. The software can be downloaded for free and its usage would enable to uniform the way laboratory errors are identified and classified, thus facilitating worldwide benchmarking activities.

## Conclusions

In a broader sense, any software that can replace human thinking could be considered a form of AI [68]. To this end, the opportunities that intelligent systems may offer to improve quality and safetyin the preanalytical phase are many and multifaceted, as summarized in Table 1. Although it seems obvious that the analytical and post-analytical phases could be the most important fields of application in laboratory medicine, a kaleidoscope of new opportunities has emerged to extend the benefits of AI to many manual labor-intensive activities belonging to the pre-analytical phase, which are inherently characterized by enhanced vulnerability and higher risk of errors. These potential applications involve increasing the appropriateness of test prescription (with computerized physician order entry or demand management tools), improved specimen collection (using active patient recognition, automated specimen labeling, vein recognition and blood collection assistance, along with automated blood drawing), more efficient sample transportation (facilitated by the use of pneumatic transport systems or drones, and monitored with smart blood tubes or data loggers), systematic evaluation of sample quality (by measuring serum indices, fill volume or for detecting sample clotting), as well as error detection and analysis. Many of these already established supports have already contributed to lightening, accelerating, improving, and even making preanalytical activities much safer; others are in advanced stages of development (particularly blood drawing robots and smart blood tubes), thus paving the way for an even better future management of the preanalytical phase.

## Dodatak

### Conflict of interest statement

All the authors declare that they have no conflict of interest in this work.
